# Generation of a mouse model of T-cell lymphoma based on chronic LPS challenge and TGF-β signaling disruption

**DOI:** 10.18632/genesandcancer.32

**Published:** 2014-09

**Authors:** Nina M. Muñoz, Lior H. Katz, Ji-Hyun Shina, Young Jin Gi, Vipin Kumar Menon, Mihai Gagea, Asif Rashid, Jian Chen, Lopa Mishra

**Affiliations:** ^1^ Department of Gastroenterology, Hepatology, and Nutrition, The University of Texas MD Anderson Cancer Center, Houston, TX, USA; ^2^ Veterinary Medicine & Surgery, The University of Texas MD Anderson Cancer Center, Houston, TX, USA; ^3^ Department of Pathology, The University of Texas MD Anderson Cancer Center, Houston, TX, USA

**Keywords:** TGF-β, β2-spectrin, Smad3, T cell lymphoma

## Abstract

Alcoholic liver disease has various manifestations: asymptomatic steatosis, alcoholic hepatitis and alcoholic cirrhosis, which substantially increase the risk for developing hepatocellular carcinoma. Transforming growth factor (TGF-β) signaling pathway is a major regulator in chronic liver diseases contributing to all liver disease progression from liver injury, inflammation and fibrosis to HCC. With the aim of generating a mouse model of alcoholic liver disease that would rapidly develop steatosis, inflammation as well as fibrosis, we formulated a regimen that combined chronic injections of low dose (2mg/kg) lipopolysaccharide (LPS) with Lieber DeCarli-based diet containing 6.7% ethanol feeding to mice with impaired TGF-β signaling through constitutive disruption of β2-spectrin and/or Smad3. Unexpectedly, the mice treated with chronic low dose LPS and fed the alcohol-containing diet developed very aggressive T-cell lymphomas to which the TGF-β mutant mice succumbed more rapidly than the wild type mice. In contrast, their liver phenotype was mild as they only developed steatosis but not hepatitis or significant fibrosis. To our knowledge, this is the first report of a mouse model of aggressive T- cell lymphoma based on chronic challenge with low dose LPS and TGF-β disruption.

## INTRODUCTION

Chronic alcohol abuse may lead to the development of alcoholic liver disease (ALD), a major cause of morbidity and mortality worldwide. ALD has various manifestations: asymptomatic steatosis, alcoholic hepatitis and alcoholic cirrhosis, which substantially increase the risk for developing HCC. The oxidative metabolism of ethanol in the liver has been extensively studied as it is the main cause of alcohol-induced liver toxicity. The production ofacetaldehyde, a toxic metabolite, the alteration of NADH:NAD+ ratio in hepatocytes and thesubsequent changes in lipid and glucose metabolism as well as the production of reactive oxygen species (ROS) are at the core of the damage that chronic alcohol consumption causes to the liver tissue [1]. Interestingly, it has been demonstrated that elevated alcohol intake also affects the permeability of the gut, which facilitates the abnormal translocation of bacteria and their metabolites, like LPS, across the gastrointestinal barrier. They can then enter into the portal circulation and further promote ethanol-induced liver toxicity [2]. One of the mechanisms for this phenomenon is the response triggered by LPS signaling through its receptor, Toll-like receptor-4 (TLR-4). In brief, TLR-4 activation in Kupffer cells, resident macrophages of the liver, and in hepatic stellate cells (HSC) results in the release of inflammatory cytokines and chemokines such as IL-1, IL-6, TNF-α and MCP-1 as well as profibrogenic cytokines like TGF- β and Platelet-derived Growth Factor (PDGF). Together, they promote hepatocyte injury and death as well as deposition of extracellular matrix components in the liver parenchyma [3].

The Transforming Growth Factor-β (TGF-β) signaling pathway is essential for normal growth and development and is often deregulated in pathological processes. Alterations in the production of TGF-β have been linked to numerous pathological conditions including liver, breast and colon cancers as well as fibrotic diseases of the liver and kidney. Furthermore, mutations in the genes for TGF-β, itsreceptors, or the intracellular signaling molecules associated with TGF-β are also important in the pathogenesis of many diseases, including liver and colon cancer [4]. TGF-β mediates its effects through a heterotetrameric receptor complex that consists of membrane-associated serine- threonine kinases. The ligand-activated receptor complex phosphorylates and activates Smad2 and Smad3, which then shuttle to the nucleus and form a complex with Smad4. Activated Smad complexes additionally recruit transcriptional co-activators, co-repressors, and chromatin remodeling factors and hence regulate the expression of a multitude of target genes. Stimulation of the TGF-β pathway can result in a variety of different effects that include cell cycle arrest in G1/S phase, induction of apoptosis, suppression of immune function, stimulation of connective tissue deposition, and maintenance of genomic stability [4]. Mishra *et al*. have previously demonstrated that disruption of the Smad adaptor protein β2-spectrin (gene *Sptbn1*) leads to disruption of TGF-β signaling mediated by Smad proteins. This effect is primarily due to mislocalization of Smad3 and Smad4 and the subsequent loss of the TGF-β-dependent transcriptional response [5]. Moreover, the same authors have shown that in contrast to wild type mice, 40% of the heterozygous β2-spectrin knock-out mice spontaneously develop HCC after 65 weeks of age [6]. Likewise, it has been demonstrated that Smad3 plays an important role in the progression of gastrointestinal cancers, and that it is a powerful tumor suppressor gene in the liver, where it plays a protective role against carcinogen-induced HCC [7].

Because many patients with steatosis never progress to steatohepatitis, the progression has been explained by a widely accepted “two-hit” theory [8]. In such model, the first hit refers to factors that promote the development of steatosis, and the second hit to those factors that facilitate the progression to steatohepatitis and more advanced stages of ALD. Previous studies from our laboratory have indeed indicated that feeding an ethanol-containing diet alone to Smad3 or β2-spectrin deficient mice, for even more than a year, does not cause ALD (unpublished data). This indicates that a “second hit” is absent in the animal model. Hence, with the aim of generating a mouse model of ALD that would rapidly develop all the key features of the human disease, i.e. steatosis, inflammation and fibrosis, we implemented a regimen of alcohol feeding combined with chronic treatment with low doses of LPS in mice with constitutive disruption of TGF-β signaling through heterozygous deletion of *Smad3* or *Sptbn1* (β2-spectrin) [6].

## RESULTS

### Disruption of the TGF-β signaling pathway accelerated the development of the T-cell lymphoma in the alcohol- and LPS-treated mice

**Figure 1:**
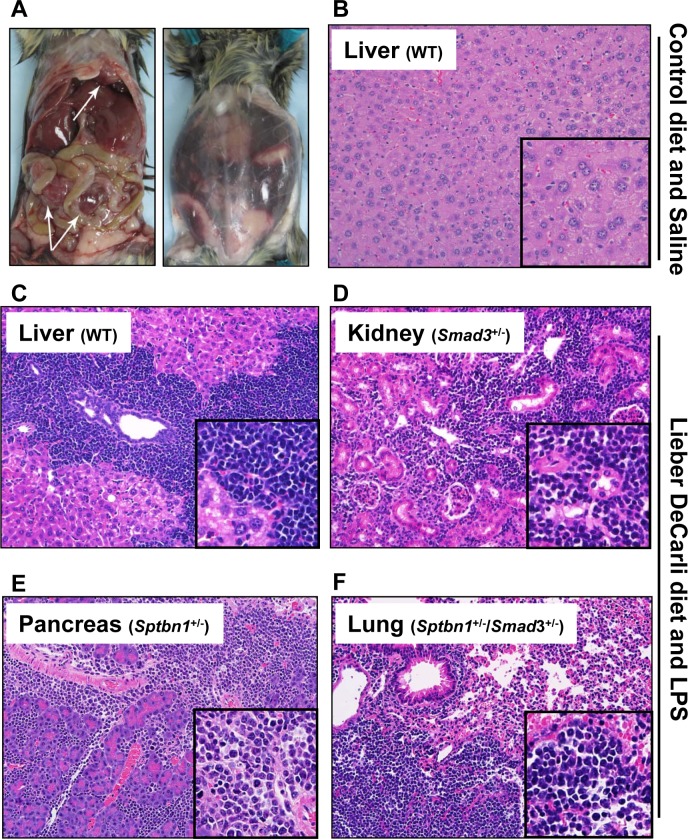
Alcohol- and LPS-treated mice develop aggressive lymphoma (A) Mice fed with a Lieber DeCarli-based diet with 6.7% ethanol and treated with intraperitoneal injections of low dose LPS (2mg/kg) develop aggressive lymphomas (arrows, left panel), accompanied by massive thoracic and abdominal hemorrhages (right panel). (B-E) The lymphoma involves non-lymphoid tissue. H&E staining with several tissues such as liver (WT) (B), kidney (*Smad3^+/−^*) (C), pancreas (*Sptbn1^+/−^*) (D), and lung (*Sptbn1^+/−^*/*Smad3^+/−^*) (E). Insets: 2× magnification of the areas selectedby dashed rectangles.

**Figure 2:**
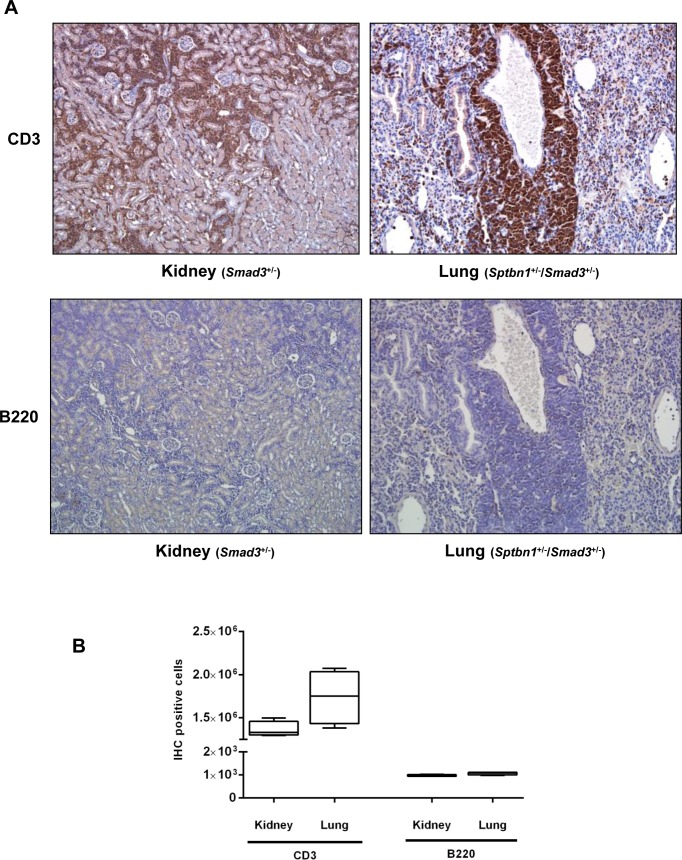
Alcohol- and LPS-induced lymphoma in TGF-β signaling pathway mutant mice is T-cell lymphoma (A) Alcohol- and LPS-treated mice develop T-cell lymphomas as demonstrated by the expression of the T-cell marker CD3, and the absence of expression of the B-cell marker B220. (B) Bar graph indicates CD3 (T-cell lymphoma) or B220 (B-cell lymphoma) positive cell number.

**Figure 3:**
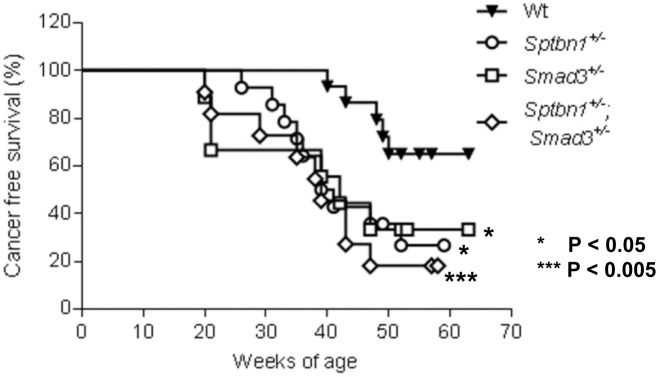
TGF-β signaling pathway impaired mice survive shorter than WT mice under alcohol- and LPS-treatment Mice with constitutive disruption of the TGF-β signaling via heterozygous deletion of β2-Spectrin (*Sptbn1^+/−^*) (n=14), Smad3 (*Smad3^+/−^*) (n=9) orboth (*Sptbn1^+/−^*; *Smad3^+/−^*) (n=11) treated with alcohol and LPS have significantly decreased cancer-free survival compared to wild type mice (n=15). *p<0.05/ ***p<0.005.

Surprisingly, TGF-β signaling pathway mutant mice fed with the Lieber DeCarli diet containing 6.7% ethanol and treated with LPS, but not wild type mice treated with the same diet or control animals, started dying 10-20 weeks after the beginning of the treatment, i.e. at 20-30 weeks of age. Specifically, by 6 months of age (26 weeks), the survival of control mice of all genotypes was 100%. In contrast, at the same timepoint the survival of ethanol and LPS-treated mice had declined to 66.7% (*Smad3^+/−^*), 92.8% (*Sptbn1^+/−^*) and 81.82% (*Smad3^+/−^; Sptbn1^+/−^*), respectively, while the survival of the wild type mice remained 100%. Pathological examination demonstrated that all the animals that died had massive hemorrhages in the abdominal and thoracic cavities. Histological examination after staining of tissue sections with Hematoxylin and Eosin (H&E) revealed that the mice succumbed because of very aggressive lymphomas that affected both lymphoid and non-lymphoid tissues such as liver, kidney, pancreas and lung (Figure 1). Analyses of the lymphocytic markers CD3 (DAKO) and B220 (BD Pharmingen) by immunohistochemistry (IHC) demonstrated that all thetumors were positive only for CD3, indicating that the cancers were T-cell lymphomas (Figure 2A). The bar graph clearly shows that the number of CD3-positive cells is markedly higher than that of cells positive for B220, marker for B-cell lymphoma in the serial slides (Figure 2B). Survival analyses carried out for 63 weeks indicated that the median survival of the TGF-β mutants oscillated between 39 to 42 weeks while it was undefined for the wild type mice (Figure 3). These results show that constitutive disruption of the TGF-β signaling pathway accelerated the development of the T-cell lymphoma in the alcohol- and LPS-treated mice and thereby it significantly impaired their survival relative to the wild type animals. Paired comparisons with the Gehan-Breslow- Wilcoxon test had p-values of 0.0007 for *Smad3^+/−^;Sptbn1^+/−^* vs. wild type, 0.0163 for *Smad3^+/−^*vs. wild type, and 0.0063 for *Sptbn1^+/−^*vs. wild type, respectively.

### Steatosis is the major phenotype in the alcohol- and LPS-treated mice

**Figure 4:**
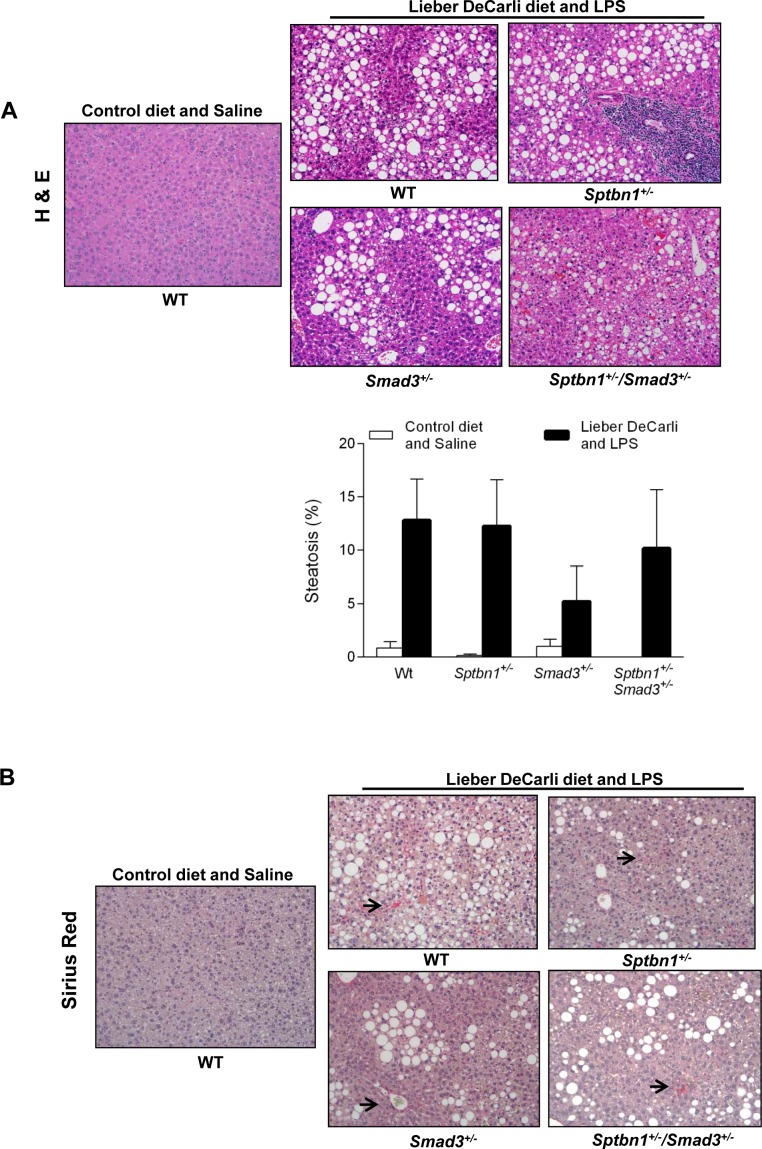
Alcohol- and LPS-treated mice develop liver steatosis but not significant liver fibrosis (A) Histological examination of the liver indicates that alcohol- and LPS-treated mice develop considerable liver steatosis compare with control mice (control diet with saline) (H&E staining and bar graph). (B) Sirius Red staining shows that alcohol- and LPS-treatment have no effect on liver fibrosis (arrow indicates Sirius Red positive cells).

Regarding the liver phenotype, staining of tissue sections with H&E as well as histochemical staining with Sirius red (for histological visualization of collagen I and III fibers) indicated that in spite of the “two hit” protocol, the animals that received alcohol in the diet along with LPS injections and that survived the longest only developed considerable degrees of steatosis, but insignificant fibrosis and no inflammation (Figure 4).

## DISCUSSION

The TGF-β signaling pathway is a potent regulator of the immune system, mainly due to its effects on antigen presenting cells and T-cells [9]. TGF-β blocks IL-2 production thus inhibiting IL-2-dependent T-cell proliferation, particularly of cytotoxic CD8+ cells. TGF-β alsoinhibits maturation of T-cells and murine models have demonstrated that abrogation of TGF-β signaling in T-cells leads to spontaneous T-cell differentiation and autoimmune diseases of the colon and lung. In hematological malignancies, resistance to the suppressive effects of TGF-β occurs mainly through decreased levels of expression of the TGF-β receptors [10]. Additionally, Smad3 protein levels are significantly reduced or even completely absent in childhood T-cell acute lymphocytic leukemia [11]. Disruption of the TGF-β signaling pathway in T-cells is therefore an important step in the development of T-cell lymphomas and leukemia due to uncontrolled proliferation of CD8+ T-cells [9].

Several mouse models of T-cell lymphoma have been previously reported, although none of them based on recurrent challenge to the immune system by an endotoxin and disruption ofthe TGF-β pathway. They have been primarily based on xenotransplantation of human lymphoma cells into immunodeficient mouse strains and treatment with Φ-irradiation or chemical carcinogens like ethylnitrosourea or nitrosomethylurea to animals with various genetic backgrounds. Genetically engineered mice prone to T-cell lymphoma such Eμ-tTA/tetO-MYC [12], Trp53^−/−^ [13] and Lck-Tert [14] have also been generated. The regimen we here devised does not produce the ALD liver phenotype we hypothesized, possibly because of prematuredeath due to lymphoma or because the mice developed resistance to the effect of LPS on the liver but not the immune system; nevertheless, the regimen resulted in a novel model of aggressive T-cell lymphoma with unique phenotypic characteristics that should be useful for further research on the biology of endogenous T-cell lymphoma. Moreover, since ethanol feeding alone does not result in early death or the development of lymphoma, we postulate that chronic LPS challenge and disruption of TGF-β signaling are the two main factors driving the development of this malignancy.

## MATERIALS AND METHODS

### Mice and Diet

All experimental procedures involving mice were approved by the Institutional Animal Care and Use Committee (IACUC) of MD Anderson Cancer Center. Wild type (n=15), *Smad3^+/−^* (n=9), *Sptbn1^+/−^* (n=14), and compound heterozygous *Smad3^+/−^; Sptbn1^+/−^* (n=11) female mice (C57BL/6) of 8-10 weeks of age were transitioned from a regular mouse chow to a Lieber DeCarli-baseddiet containing 6.7% ethanol (BioServ, #F1258SP) [6]. After 2 weeks, the animals started a regimen of intraperitoneal injections of PBS only or 2mg/kg LPS from *E. coli* (Sigma Aldrich #L2654) diluted in PBS three times a week. For control, a group of mice of the same ages and genotypes [wild type (n=7), *Smad3^+/−^* (n=6), *Sptbn1^+/−^* (n=7), and *Smad3^+/−^; Sptbn1^+/−^* (n=5)] received an isocaloric diet without alcohol (Bioserv #F1259SP) and 3 weekly injections of normal saline solution. The animals were closely monitored for symptoms of physical distress or illness.

### Immunohistochemical analysis

Tissues were fixed in 10% buffered formalin, and embedded in paraffin. Five micron paraffin sections were stained with the CD3 antibody (1:100–1:200; DAKO,CA) or B220 antibody (20ug/ml, BD Pharmingen, CA), then DAB staining was carried out according to manufacturer instructions (Vectastain ABC kit, Vector Labs). Hematoxylin and eosin staining were used for counterstaining. Sirius Red staining was performed with Direct Red 80 (Sigma-Aldrich). After the slides were stained, they were scanned and analyzed by an ACIS III image analyzer (DAKO, CA).
